# Supplementary material for: Efficient long-range conduction in cable bacteria through nickel protein wires
Source: Nat Commun. 2021 Jun 28;12:3996. doi: 10.1038/s41467-021-24312-4 (PMC8238962; doi:10.1038/s41467-021-24312-4)
Supplement: Supplementary file 1 — Supplementary information [file 41467_2021_24312_MOESM1_ESM.pdf]

# Supplementary information for

# Efficient long-range conduction in cable bacteria through nickel protein wires

Henricus T. S. Boschker, Perran L.M. Cook, Lubos Polerecky, Raghavendran Thiruvallur Eachambadi, Helena Lozano, Silvia Hidalgo-Martinez, Dmitry Khalenkow, Valentina Spampinato, Nathalie Claes, Paromita Kundu, Da Wang, Sara Bals, Karina K. Sand, Francesca Cavezza, Tom Hauffman, Jesper Tataru Bjerg, Andre G. Skirtach, Kamila Kochan, Merrilyn McKee, Bayden Wood, Diana Bedolla, Alessandra Gianoncelli, Nicole M.J. Geerlings, Nani Van Gerven, Han Remaut, Jeanine S. Geelhoed, Ruben Millan-Solsona, Laura Fumagalli, Lars-Peter Nielsen, Alexis Franquet, Jean V. Manca, Gabriel Gomila, Filip J. R. Meysman

**This file includes:**

Supplementary Notes

Supplementary Figures 1 to 16

Supplementary Table 1

**Other Supplementary information for this manuscript include the following:**

Supplementary Movie 1

Excel files with Supplementary Data 1 and 2

## Supplementary Notes

### Note 1: Additional results and discussion of the ToF-SIMS analysis

The principal results of the ToF-SIMS analysis are described in the main text. Here we provide additional experimental results and discussion to support the conclusions drawn in the main text.

*Fragment identification*. In total 71 fragment ions were identified in positive mode and 173 in negative mode in the ToF-SIMS runs of the fiber sheaths. Identification was based on the exact mass in ToF-SIMS analysis and was checked for identity and purity with the Orbitrap-SIMS runs. For transition metals and S, the isotopic mass distribution was verified. For Ni, the four main isotopes ($^{58}$Ni, $^{60}$Ni, $^{61}$Ni and $^{62}$Ni) were found with the expected isotopic abundance, whereas the minor isotope $^{64}$Ni showed mass interference from a low amount of Zn. For Cu, both isotopes ($^{53}$Cu and $^{65}$Cu) showed the expected abundance. For Fe, the two main Fe isotopes ($^{54}$Fe and $^{56}$Fe) showed the expected abundance, whereas the minor Fe isotopes ($^{57}$Fe and $^{58}$Fe) showed mass interference from an unknown source and Ni respectively. Zn counts were low and not reliable as the main isotope ($^{64}$Zn) showed mass interference from $^{64}$Ni. Other transition metals found in metalloproteins like Mn, Co, Mo and W were not detected or had very low counts. The main isotopes of Sulfur ($^{32}$S and $^{34}$S) also showed the expected abundances, whereas the minor isotopes $^{33}$S and $^{36}$S showed mass interference from an unknown source. Assignment of the identified fragment ions to biochemical components of intact cable bacteria and the fiber sheath was based on literature[14–20] and specific depth profiles (see Supplementary Data 1 and 2, Fig. 1, and Supplementary Figure 1).

*Transition metals.* The ToF-SIMS depth profiles of Ni and Fe are discussed in detail in the main text. Cu showed a peak at the first data point of the depth profile in both the fiber sheaths and the intact cable bacteria (Fig. 1D, Supplementary Figure 1A-B, Supplementary Figure 11), though with a highly variable count number. This surface peak and its variability between samples was consistent with our LEXRF analysis (Fig. 3), which also showed a highly variable Cu signal. However, Cu counts from STEM-EDX analysis and the majority of the ToF-SIMS depth profiles in the fiber sheaths were low compared Ni counts. This suggests that Cu is not present in high concentrations, but likely derives from an variable impurity that adsorbs to the sample surface at some stage during sample preparation (either cable bacteria collection, fiber sheath extraction procedure) or analysis.

*Polysaccharide fragments.* As discussed in the main text, the basal polysaccharide layer (Fig. 1D and 1E) most likely consists of peptidoglycan, which consists of an acidic amino sugar backbone interconnected by short peptides[21]. We therefore expected to find fragments that contain both oxygen and nitrogen, as derived from the amino sugar backbone of peptidoglycan. To verify that we can detect peptidoglycan specific fragments among these O- and N rich fragments, we performed ToF-SIMS analysis of four protein and polysaccharide reference samples: Bovine Serum Albumin (BSA), starch, pectin, and peptidoglycan (Supplementary Figure 8). Although the mass spectra of these reference samples showed major differences in both negative and positive mode (Supplementary Figure 8), we could not detect any peptidoglycan specific fragments in the fiber sheaths. Moreover, fragments that contained both nitrogen and oxygen showed either the same depth profile as amino acid (protein) derived fragments (i.e. an initial peak at the start of the depth profile), or were specific for nucleic acids that showed low counts and variable depth profiles (Supplementary Data 1 and 2). The latter suggests that some residual DNA/RNA remains associated with the fiber sheaths after extraction. The lack of peptidoglycan specific

fragments is most likely due to the abundance of protein in the fiber sheath, which leads to high signals of amino acid derived fragments that mask fragments derived from peptidoglycan. Further work is needed to confirm that the polysaccharide layer is indeed made of peptidoglycan.

*P-containing fragments.* The fiber sheaths also showed high counts of various P-containing fragment ions in both positive and negative mode (Supplementary Data 1 and 2) with variable depth profiles between runs. The likely source of these P-containing ions is the poly-phosphate granules that are commonly found in cable bacteria[22]. Likely, the poly-phosphate granules are incompletely removed during the fiber sheath extraction procedure, and they comprise the particles that are seen in the interior of fiber sheaths during STEM 3D-tomography (Fig. 1A).

*S and Ni containing fragments in negative mode.* Negative mode ToF-SIMS depth profiles showed a subsurface peak of various S-derived anions ($^{32}S^-$, $^{34}S^-$, $SH^-$ and $S_2^-$) at approximately the same position as the Ni peak in positive mode (Fig. 1D and 1E, Supplementary Figure 1, Supplementary Data 2). In addition, a substantial number of Ni-containing fragments were found in negative mode that also showed this distinct subsurface peak (Fig. 1D, Supplementary Figure 1, Supplementary Data 2). The most prominent Ni containing fragments were a series of $Ni_xS_y^-$ cluster ions (x = 1 to 6, y = x +/- 1, both $^{58}Ni$ and $^{60}Ni$ isotopologues were detected, Supplementary Figure 9). Additionally, we found $NiC_xN_x^-$ (x = 1 or 2) and $NiCSN^-$, and even a small but detectable $Ni^-$ signal (Supplementary Data 2). All these Ni-containing fragments showed a similar sharp subsurface peak in the depth profile as found for Ni in positive mode (Fig. 1E). The observed NiS clusters are most likely formed in the ion plume of the ToF-SIMS rather than being natively present in the fiber sheath[23–25]. As a control, we analyzed an artificial mixture of protein (BSA) and freshly precipitated mineral NiS, and we indeed observed the same $Ni_xS_y^-$ cluster ions (Supplementary Figure

9D). Still, due to an effect called self-focusing[23], the detection of NiS clusters suggests that Ni and S must be present in close proximity in the fiber sheath (lateral (XY) within <0.5 nm, depth (Z) within <2 nm), otherwise they would not be formed in the ToF-SIMS ion plume. This observation is hence in agreement with the presence of a S-ligated Ni cofactor in the fiber sheaths.

We also found a number of organic S fragments in negative mode ToF-SIMS spectra (Fig. 1E, Supplementary Figure 1, Supplementary Data 2). Most of these fragments showed a similar depth profile as $S^-$ with a sharp subsurface peak on top of a general S signal, and so again, we cannot exclude that they were formed during ToF-SIMS analysis. However, two fragments ($C_2S_2^-$ and $C_2S_2H^-$) showed specific depth profiles, with the subsurface peak much more pronounced than for $S^-$ (Fig. 1E). This suggests that these two fragments originate directly from the fiber sheath. These fragments could indicate that the fiber proteins are rich in disulfide bridges (C-S-S-C) or they could be derived from the Ni/S group. Fiber sheaths display an exceptional chemical resistance, as they remain their integrity and conductivity after SDS and EDTA treatments, and this resistance could be aided by protein disulfide bridges.

*Sputtering depth calibration.* Sputtering depth was calibrated by *in-situ* AFM height measurements at three different times (Supplementary Figure 2): before sputtering, just after the Ni or S peak appeared, and finally just after the carbohydrate peak appeared. Sputtering depth was approximately linear with sputtering time, although the initial protein layer seemed to sputter somewhat faster (Supplementary Figure 2). A representative example of the resulting AFM height maps is shown in Supplementary Figure 10, where sputtering was stopped at 57 sec (i.e. just after the Ni peak had emerged). There was considerable lateral variation in sputtering depths for a given sputtering time as seen in transects across the central cell area (Supplementary Figure 10D) and at the cell junctions (Supplementary Figure 10C).

As the junctions are higher than the cell areas (e.g. Supplementary Figure 10A), the shading in sputtering seen at the cell junctions probably due the angled Ar-cluster sputter beam hitting the side of the cell junction facing the beam at a higher angle whereas the opposite side is hit much less. This lateral variation in sputtering rate together with the left-over cytoplasm content and the presence of the cart-wheel structure at the junctions leads to smearing of the ToF-SIMS depth profiles. This explains why only the first fiber sheath layer is clearly seen in the ToF-SIMS depth profiles.

**Note 2: Additional results and discussion of the SDM analysis**

The key results of the Scanning Dielectric Microscopy (SDM) analysis are described in the main text. Here we describe additional results that strengthen the conclusions obtained. The finite element model we used is the same as presented earlier[5–7] except that here, fibers have a core-shell structure with a conductive core and an insulating outer shell. The whole simulation domain (which encompasses the ellipsoid fiber and the surrounding space) is cylindrical with height 34 μm and radius 17 μm. Insulating boundary conditions are assumed on the lateral and top borders of the simulation domain. A cross-section of the electric potential distribution obtained from the model calculations is shown in the right insert in Fig. 5C. At the frequency of the calculations (2 kHz) and for the parameters in Fig. 5, the electric potential is real, and hence, the phase of the electric potential is constant in space. The length of the fiber in the model is $L = 1$ μm. It has been shown previously that for $L > 100$ nm the force acting on the tip is independent from the fiber length[6].

Supplementary Figure 12A shows experimentally derived capacitance gradient cross-section profiles corresponding to SDM images (similar to the one shown in the left insert in Fig. 5B of the main text) acquired at different tip-substrate distances (continuous lines,

ranging from z=60 nm to z=240 nm). The profile at z=66 nm is shown in Fig. 5C of the main text. The dashed lines represent the result of the theoretical calculations with the model described above for the same fiber parameters as those use in Fig. 5 of the main text (h=42 nm, w=87 nm, d=12 nm, $\varepsilon_s = \varepsilon_c=3$, $\sigma_s=0$ S/cm, $\sigma_c=20$ S/cm). The tip-substrate distances have been determined from a capacitance gradient approach curve measured on a bare part of the substrate (Supplementary Figure 12B, symbols) following procedures previously reported[5]. The calculated capacitance gradient profiles nicely reproduce the experimental ones with no adjustable parameter. At all distances, the theoretical (and experimental) electric force phase contrast is zero (not shown). Similar conclusions are reached if we analyze the capacitance gradient approach curves measured on the substrate and on the fiber (pink and black thick lines in Supplementary Figure 12B). A least square fitting of the model curves to the experimental curve for $\varepsilon_s = \varepsilon_c=3$ and $\sigma_s=0$ S/cm, $\sigma_c=20$ S/cm gave d=12±2 nm (red continuous line in Supplementary Figure 12B), in agreement with the value obtained from the capacitance gradient cross-section profile analysis. The sensitivity of the capacitance gradient profiles on the thickness of the insulating shell is shown by the dashed lines in Supplementary Figure 12B for the approach curves and in Supplementary Figure 12C for the profiles at z=66 nm. In these figures we also compare the predictions corresponding to an homogeneous conductive model, corresponding to d=0 nm (or to $\sigma_s = \sigma_c=20$ S/cm, dark grey line), and to an homogeneous insulating model, corresponding to d=h/2 (or $\sigma_c=0$ S/m, light grey line). In all cases we assumed a protein composition of all layers, $\varepsilon_s = \varepsilon_c=3$. The pure conductive model overestimates the force acting on the tip, while the pure insulating model underestimates it.

We have considered other possible sets of electric parameters for the fiber to explore alternative interpretations of the SDM results. Figs. S13A and S13B show, respectively, the contrast values of the amplitude (in zF/nm) and phase of the electrical force for the tip at the center of the fiber at a distance z=66 nm from the substrate as a function of the conductivity

of the core in the range from $\sigma_c = 10^{-9}$ S/m (insulator) to $10^3$ S/m (conductor). The dielectric constants have been fixed to $\varepsilon_s = \varepsilon_c = 3$ (proteins). The thickness of the insulating layer has been varied from d=0 nm to d=20 nm. The calculations were done for the frequency of the experiments (2 kHz). The amplitude as a function of the core conductivity displays two plateaus for low and high conductivities, separated by a transition region, which tends to show a third plateau not fully displayed. The phase shows a minimum for every transition region mentioned above. Such behavior is characteristic of materials with (equivalent) permittivities showing real (dielectric) and imaginary (conductive) parts. An analytical expression for the equivalent homogeneous permittivity of the core-shell cylinder in a non-uniform electric field cannot be derived, but the behavior observed is qualitatively similar to the one corresponding to a core-shell spherical particle in a uniform electric field[26]. For a given measured capacitance gradient contrast (grey band in Supplementary Figure 13A), one can obtain couples of values for the shell thicknesses and core conductivity that match[5,27]. For instance, we observe that the solution found above for the shell thickness (d=12 nm) is valid for $\sigma_c > 10^{-2}$ S/m (and, in particular, for $\sigma_c = 2 \cdot 10^3$ S/m as employed here). Other couples of values are for instance d=5 nm and $\sigma_c = 5 \cdot 10^{-6}$ S/cm or d=0 nm, $\sigma_c = 7 \cdot 10^{-7}$ S/cm, among others. For these values the same capacitance gradient profile (matching the experimental one) is obtained, as shown in the Supplementary Figure 13C. However, the different couples of values predict different electrical phase contrast profiles (see Supplementary Figure 13D). Only the couple of values corresponding to the solution reported in the main text (d=12 nm and $\sigma_c > 10^{-2}$ S/m) predicts a null phase contrast, as seen in the experimental results.

We have also considered a homogeneous dielectric model, with no conductivity, and analyzed the (equivalent) homogeneous dielectric constant that the fiber should have to explain the experimental results. A least square fitting of the calculated capacitance gradient approach curves for different dielectric constants to the experimental curve measured on the

fiber gives $\varepsilon_s = \varepsilon_c = 11\pm3$ (see Supplementary Figure 14A). For this equivalent dielectric constant value the constant height capacitance gradient profiles at the different tip substrate distances adequately reproduce the experimental ones (Supplementary Figure 14B). The phase contrast for this model is zero (not shown). However, a problem is that the equivalent dielectric constant value found ($\varepsilon_s = \varepsilon_c = 11\pm3$) is much larger than values obtained, with the same technique, on other (dry) bio-samples made of lipids ($\varepsilon=2$)[28], proteins ($\varepsilon=3-5$)[27,28,6,7] and even nucleic acids ($\varepsilon\sim8.5$)[5,27] (see Supplementary Figure 14C). Our composition data all suggest that the fibers are made primarily of protein. During ToF-SIMS analysis of fiber sheaths, some fragments derived from residual RNA or DNA were detected, but these were not associated with the initial fiber protein layer (see Additional results and discussion of the ToF-SIMS analysis) and it therefore seems unlikely that an isolated fiber as analyzed here by SDM would contain major amounts of DNA. For this reason, the pure dielectric model is discarded in favor of the conductive core-shell model.

We have also analyzed the homogeneity of the fiber electrical properties along its longitudinal direction in view of the slight variations of the electric contrast observed in the SDM images along the fiber (see left insert in Fig. 5C of the main text). To this end we considered the core-shell geometrical model that accounts for the observed (tiny) height variations of the fiber height (±5 nm)[29] shown in Supplementary Figure 15A. Examples of calculated longitudinal and transversal electric potential distributions are shown in figs. S15B and S15C, respectively. The tip and electrical parameters of the fiber are the same as those used in the calculations of in Fig. 5 of the main text, except for the shell thickness, which here is d=10±2 nm. The slightly smaller value obtained, as compared to d=12±2 nm for the cylindrical model, is due to the tip convolution effects included in this geometrical model (see Supplementary Figure 15D), as discussed elsewhere[29]. Supplementary Figure 15E shows a calculated constant height SDM image at z=66 nm corresponding to the region enclosed by

the dashed rectangle in the insert (which corresponds to the image in the left insert in Fig. 5C of the main text). The calculated SDM image is nearly identical to the experimental one, a fact that is further evidenced by comparing the capacitance gradient cross-section profiles calculated on the hills and valleys of the image with the experimental ones (figs. S15F and S15G, respectively). This result shows that, to a good approximation, the electrical properties of the fiber are homogeneous along its length.

Finally, we have also analyzed the electric properties of the fibers when they are still embedded within the fiber sheath by SDM. Supplementary Figure 16A shows an AFM topographic image of a fiber sheath. Figs. S16B and S16C show, respectively, topographic and constant height SDM images acquired in the area enclosed by the dashed rectangle in Supplementary Figure 16A. The corresponding height and capacitance gradient profiles along the dashed lines in figs. S16B and S16C are shown in figs. S16D and S16E, respectively (black lines). The geometry of the tip has been calibrated with a capacitance gradient approach curve acquired on the substrate (not shown) giving R=26±2 nm, θ=22±3°, $C'_{offset}$=109±3 zF/nm. The sample geometry has been reconstructed by using a topographic reconstruction procedure[29], as above (see Supplementary Figure 16D). For simplicity, we have considered an equivalent homogeneous dielectric model characterized by an (equivalent) dielectric constant $\varepsilon_{sheath}$. An example of a calculated electric potential distribution is shown in Supplementary Figure 16F for a tip-substrate distance z=155 nm and $\varepsilon_{sheath}$=7. Supplementary Figure 16E shows calculated constant height capacitance gradient profiles along the dashed line in Supplementary Figure 16C for different values of $\varepsilon_{sheath}$ (thin lines). The experimental values on the hills (corresponding most likely to fiber positions) agree with the calculated ones for $\varepsilon_{sheath}$~7−11 (except the first hill that gives a somewhat larger value). These values are in reasonable agreement with those found for the isolated fiber when the equivalent dielectric model is considered ε~11±3. This result implies that the electrical properties

obtained for the fibers when within the fiber sheath are consistent with those obtained on isolated fibers. Similar results are expected for a fiber sheath model that included its internal structure, composition and electrical properties (e.g. conductive core-shell model for the fibers), as the one sketched in Fig. 5A of the main text. However, obtaining quantitative predictions from SDM measurements for such complex model involving buried structures lies outside the current capabilities of SDM[30] since too many unknowns are present in the model (e.g. fiber position within the fiber sheath, number of fibers present within the polysaccharide layer, thickness of the polysaccharide layer, etc.).

**Note 3: Calculations for estimating the contribution of the fibers to the fibers sheath, the distance between Ni atoms in the conductive fibers and electron tunneling times.**

Electron hopping rates between redox sites in metalloproteins decreases logarithmic with the distance between the redox sites[31]. Given the relatively low Ni content of the fiber sheaths of 0.017 and 0.034 Atm% as determined by STEM-EDX for two sample batches (Supplementary Table 1), we tried to estimate what the inter atomic distance of the Ni atoms would be. At the cell area thick fiber sheaths are ca. 4 μm wide and 117 nm high (this study) and contain on average 60 parallel fibers[2] that each contain a conductive core of 26 nm diameter (this study). From this we calculate that the conductive fiber cores explain only 7% of the cross-section area of the fiber sheath. If we do the same calculations for the total 50 nm fiber with that includes the non-conductive outer shell, we arrive at a 25% contribution from the fibers to the fiber sheath. Assuming that the conductive fiber core is made of protein with a density of 1.4 $g \cdot cm^{-3}$ (density from[32]) and that Ni is concentrated in the conductive core (this study), we estimate that the Ni concentration in the conductive fiber core is between 0.24 and 0.54 Atm% or 260 and 580 $\mu Mol \cdot cm^{-3}$, which are substantial concentrations. Further

assuming that Ni is homogenously distributed in the core of the fibers, this Ni concentration in the fiber core leads to an interatomic Ni distance of 1.4 – 1.9 nm, which is within the range as found for metalloproteins involved in electron transfer[31].

We then used these estimates of interatomic Ni distances to calculate electron tunneling times needed to support the high average normalized currents in fiber sheaths of 28 nA (calculated from the standard SDS+EDTA extraction data in Fig. 4D). Estimated tunneling times are 50 – 90 nsec, which is possible but on average however approximately a factor 10 faster than expected for metalloproteins with similar hopping distances[31]. It seems therefore unlikely that long distance electron redox hopping through the Ni/S-group can on its own support the high conductivity of the fibers in cable bacteria (see Discussion in the main text).

## Supplementary figures and tables

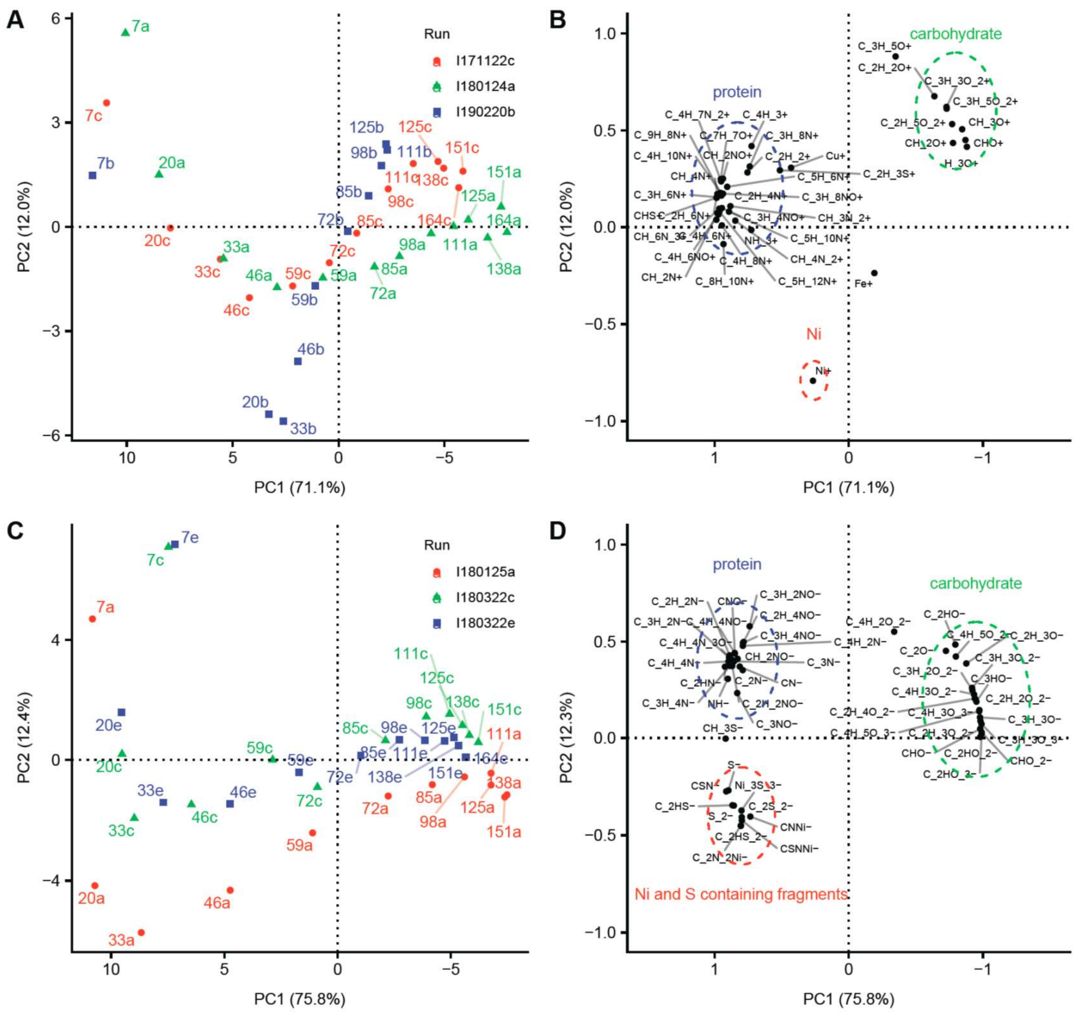


**Supplementary Figure 1.** Principal Component Analysis (PCA) of ToF-SIMS depth profiles for a selection of ion fragments recorded from fiber sheaths of cable bacteria. ToF-SIMS data were collected in both positive (A, B) and negative modes (C, D), and the results of three replicate depth profiling runs are shown. Counts for fragments were normalized and centered. Panels (A, C) show the scores plot (labels indicate sputtering time in seconds; colors denote separate runs). Panels (B, D) show the loading plots of the fragments. Only the data until the peak carbohydrate-derived fragments at 125-164 seconds of sputtering time were retained (representing the first half of the double-folded fiber sheath).

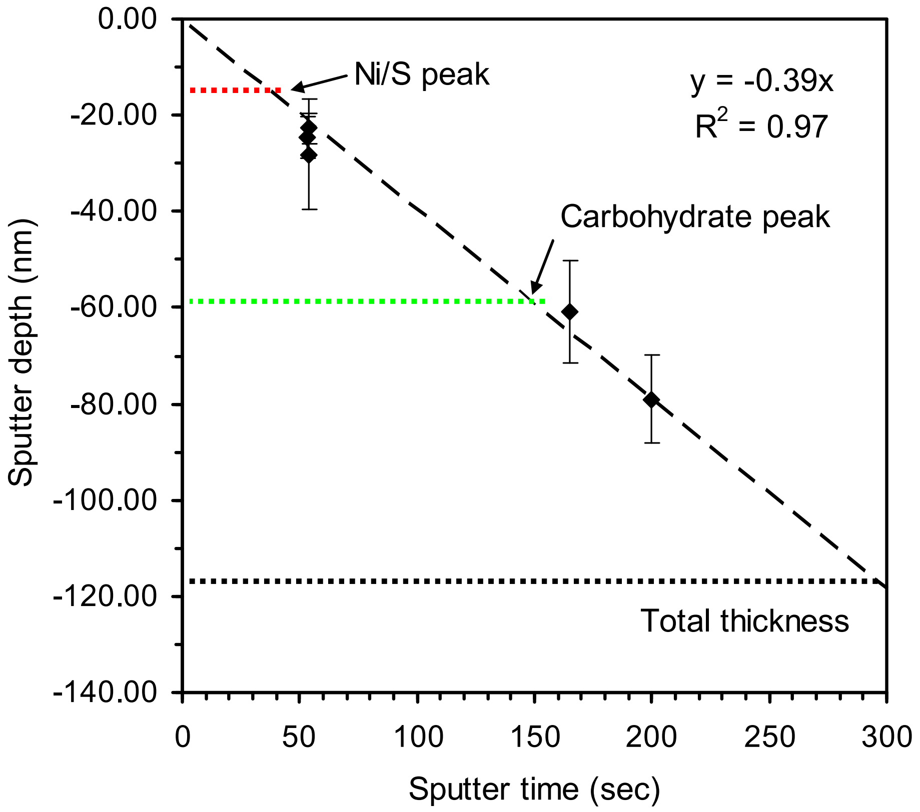


**Supplementary Figure 2.** Calibration of sputtering depth as a function of sputter time during ToF-SIMS analysis. The in-situ AFM within the ToF-SIMS instrument was used to record height images at three different times (start (sputter time = 0 sec), just after the Ni or S peak (ca. 50 sec) and just after the carbohydrate peak (ca. 160 and 190 sec)). Sputtering depth (black diamonds, average +/- SD) showed a linear relation with sputtering time (dashed line = regression line through origin), though the initial protein layer seemed to sputter somewhat faster. The total thickness of the double-folded fiber sheath amounts to 117 ± 10 nm (determined within the middle of cells at t = 0 sec; black dotted line) and is well in agreement with previous independent AFM imaging [2]. The average position of the Ni/S peak (15 ± 3 nm) and the carbohydrate peak (59 ± 6 nm) are indicated (red and green dotted lines respectively, N = 6 from 3 positive and 3 negative depth profiles in Supplementary Figure 1).

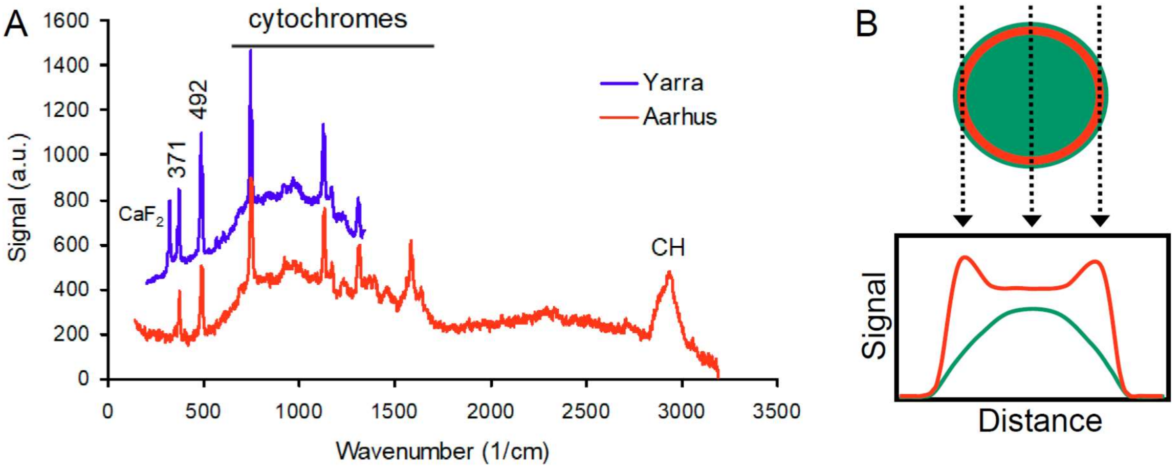


**Supplementary Figure 3.** (A) Green-laser Raman spectra of intact cable bacteria from additional sediments also show the characteristic low-frequency bands at 371 and 492 $cm^{-1}$. Spectra are from ca. 1 μm wide freshwater cable bacteria (Aarhus pond sediment, intact living filaments in glass micro-chambers, average background-corrected spectrum) and ca. 1 μm wide brackish cable bacteria (Yarra River sediment, intact filaments air-dried on a $CaF_2$ cover, average raw spectrum). (B) Interpretation of the Raman signal distribution in the scan across an individual cable bacterium in Fig. 2B. General components of a bacterial cell such as proteins and CH are more-or-less evenly distributed in the cross section (green filled circle), whereas the Ni/S group is only found in the periplasmic space (red open circle). In the depth integrated Raman signals, this leads to a unimodal distribution for Raman bands from the general components and a bimodal distribution for the two low frequency Ni/S bands with a peak at both edges of the filament.

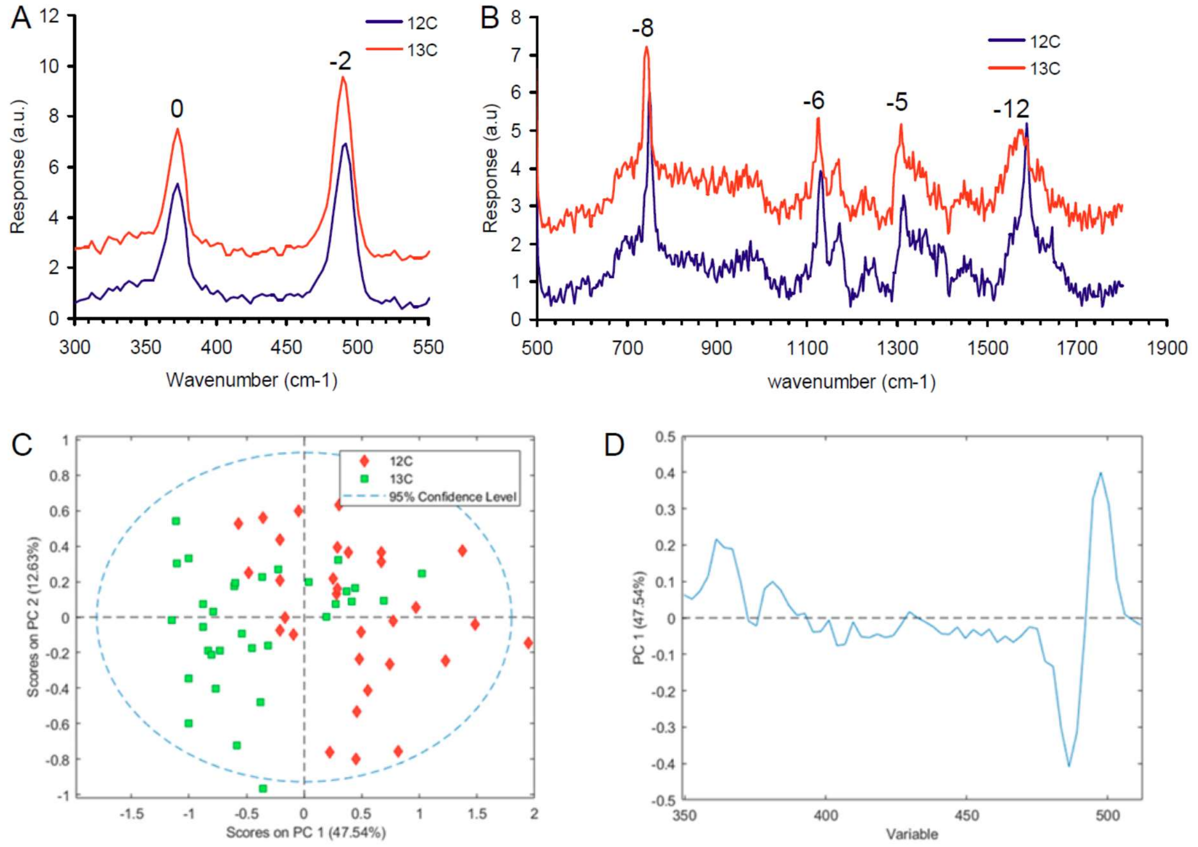


**Supplementary Figure 4.** Effect of $^{13}C$ labelling on the green-laser Raman spectra from intact cable bacteria. A) The low-frequency bands either do not shift (371 $cm^{-1}$ band) or are only slightly affected (491 $cm^{-1}$ band shifts to 489 $cm^{-1}$) by the labelling. B) The characteristic cytochrome bands clearly shift to lower values suggesting that cable bacteria filaments were highly labelled with $^{13}C$. The average of N = 32 spectra is shown for both unlabeled control and $^{13}C$ treatments and shifts in wavenumbers are indicated. C) PCA analysis of the 350 to 550 $cm^{-1}$ region of the spectra containing the low-frequency bands shows a difference between the labelled versus unlabeled treatments. D) Variable (wavenumber) loadings on the first PCA axis show that only the second lower band at 492 $cm^{-1}$ shifted slightly to lower values in the labelled spectra.

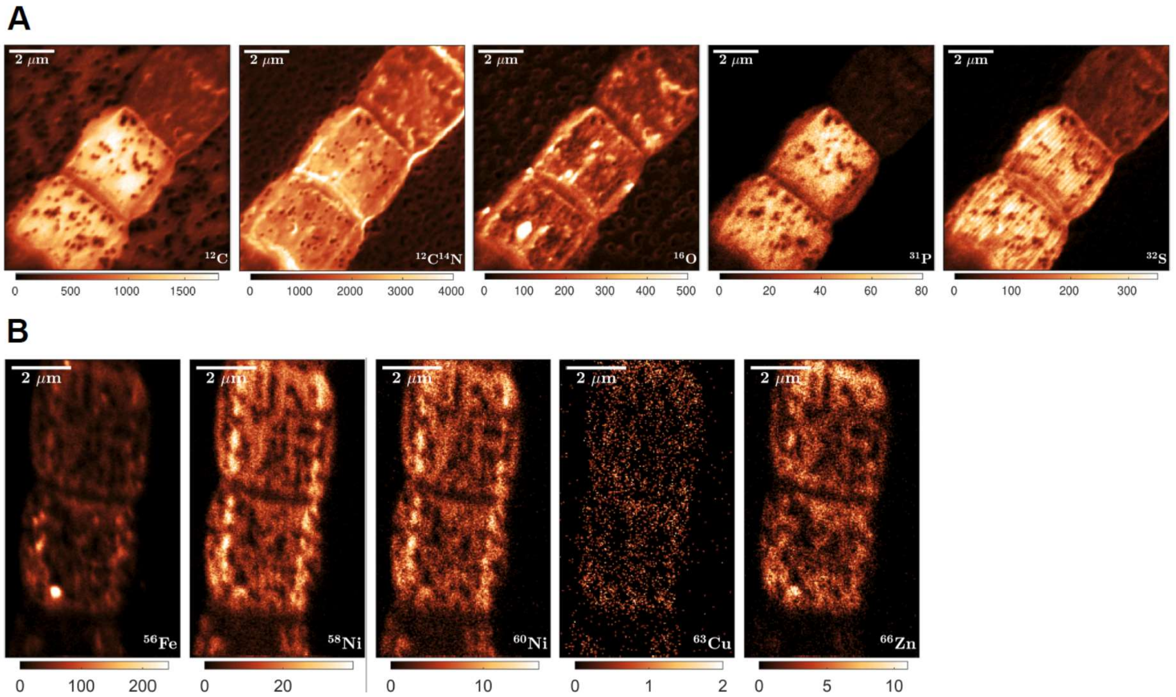


**Supplementary Figure 5.** NanoSIMS images of A) negative ions and B) positive ions of metals from single fiber sheaths deposited on a gold-coated polycarbonate filter. In A) only the first 100 planes of analysis were selected as this most clearly showed the fibers structure in the $^{32}S$ image. In B) the first 50 planes were selected as this showed the fibers most clearly in the $^{58}Ni$ image. Independent replica's (N = 2) yielded similar results.

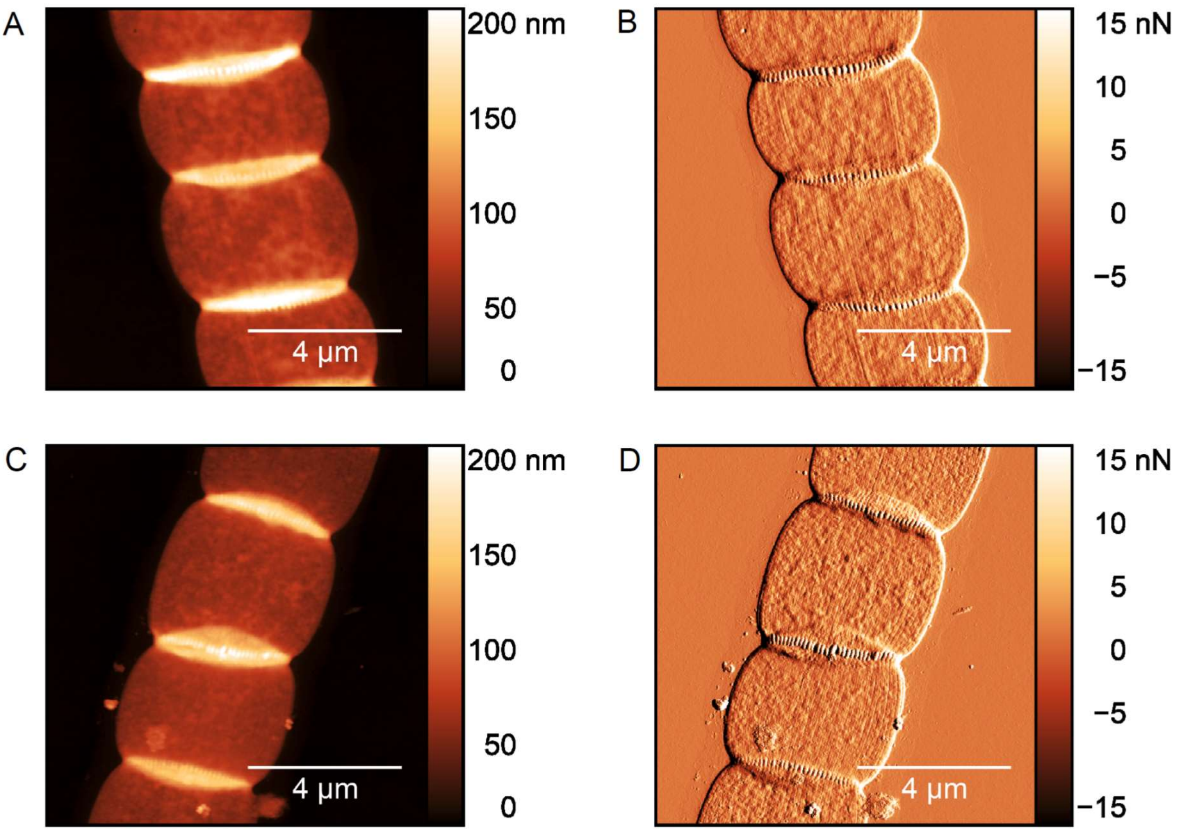


**Supplementary Figure 6.** Atomic Force Microscopy (AFM) images of fiber sheaths as extracted with the standard protocol (A and B, SDS+1 mM EDTA for 10 min) and the high EDTA treatment to remove Ni (C and D, SDS+50 mM EDTA for 10 min). Shown are the height (A,C) and peak force error (B,D) data. The parallel fiber structures are retained after high-concentration EDTA extraction. Independent replica's (N = 2) yielded similar results.

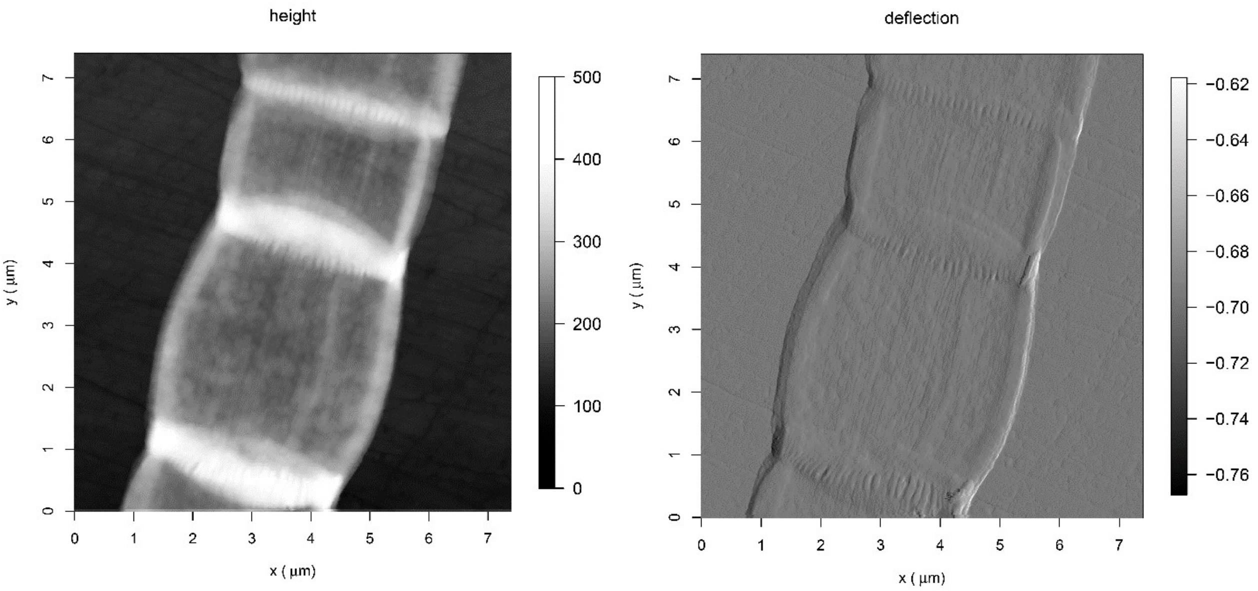


**Supplementary Figure 7.** AFM-IR height (in nm) and deflection maps of extracted fiber sheath showing the parallel fibers.

A

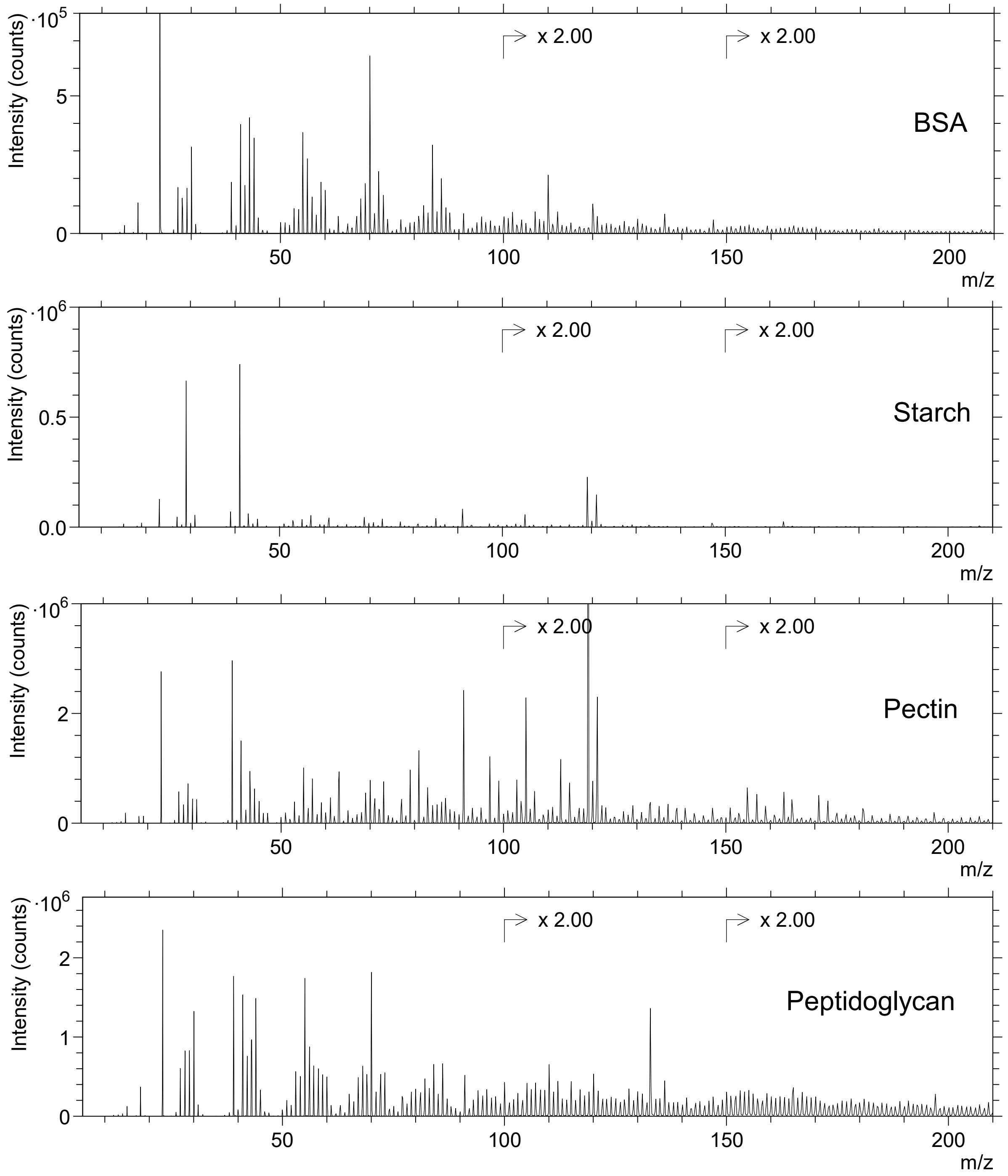

Intensity (counts)
·10^5
5
0
x 2.00
x 2.00
BSA
50
100
150
200
m/z
Intensity (counts)
·10^6
0.5
0.0
x 2.00
x 2.00
Starch
50
100
150
200
m/z
Intensity (counts)
·10^6
2
0
x 2.00
x 2.00
Pectin
50
100
150
200
m/z
Intensity (counts)
·10^6
2
1
0
x 2.00
x 2.00
Peptidoglycan
50
100
150
200
m/z


352

B

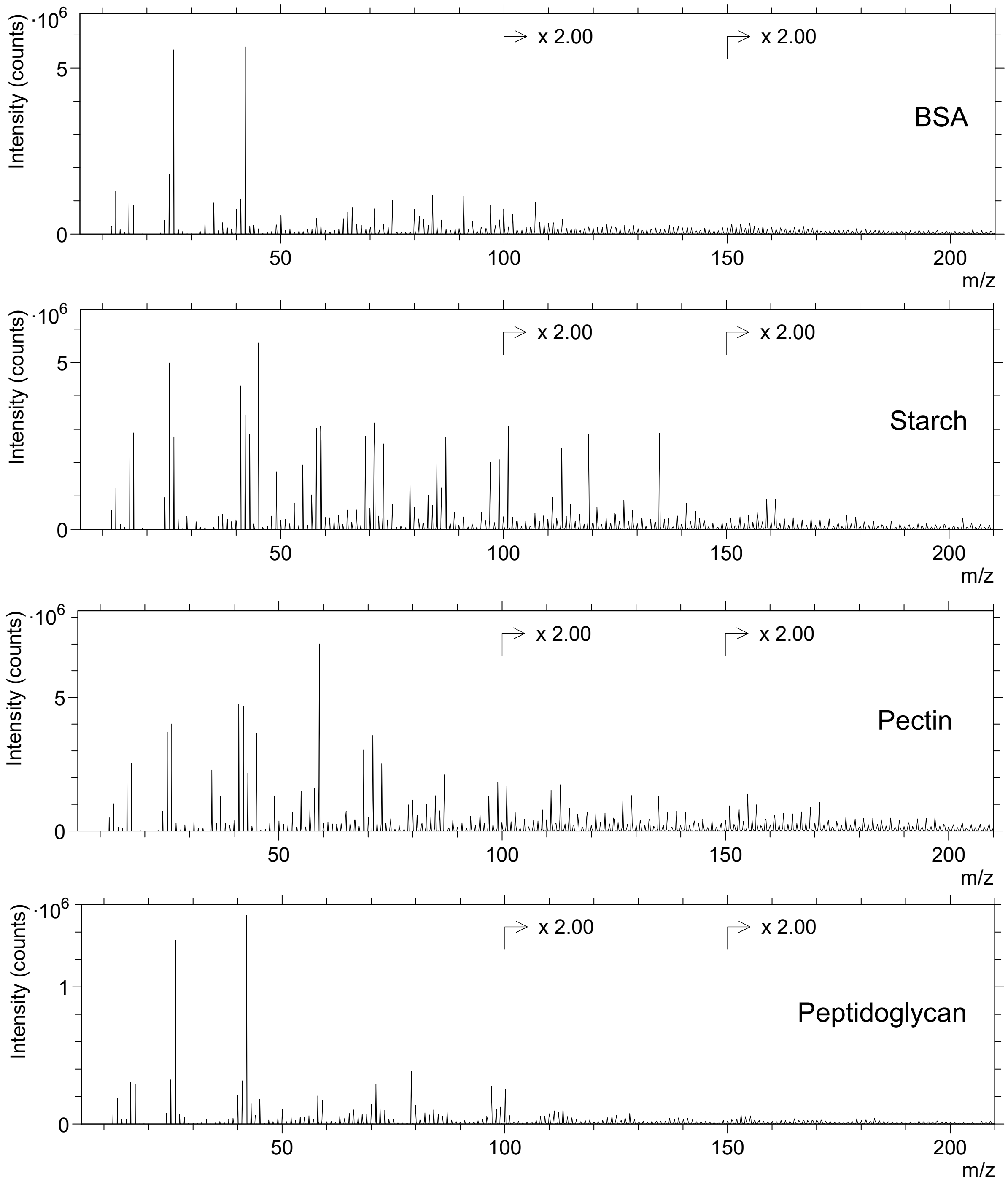


**Supplementary Figure 8.** ToF-SIMS positive (A) and negative (B) mode mass spectrum of additional protein and polysaccharide reference samples analyzed to aid in the interpretation of the fiber sheath mass spectra.

A

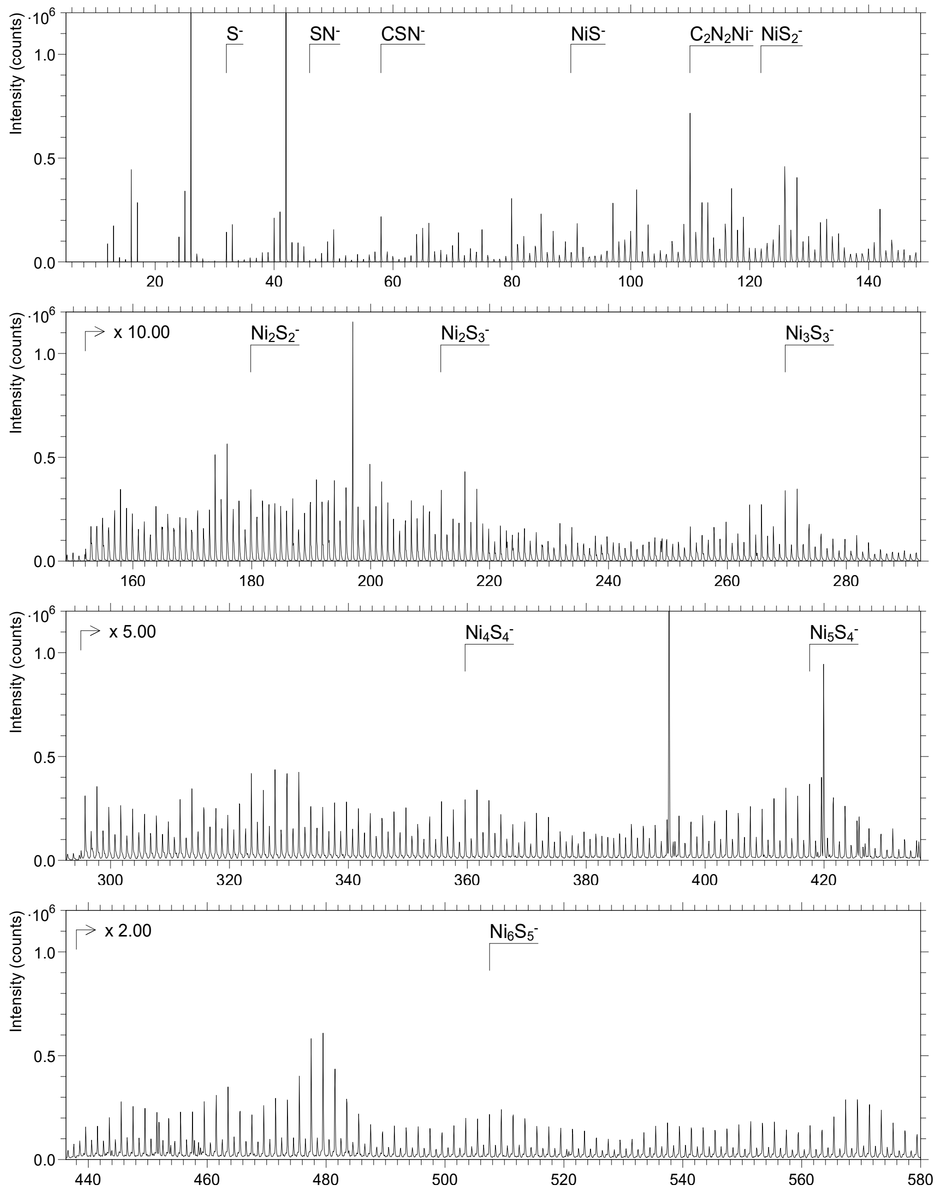
Intensity (counts)
·10^6
S⁻
SN⁻
CSN⁻
NiS⁻
C2N2Ni⁻
NiS2⁻
x 10.00
Ni2S2⁻
Ni2S3⁻
Ni3S3⁻
x 5.00
Ni4S4⁻
Ni5S4⁻
x 2.00
Ni6S5⁻

B

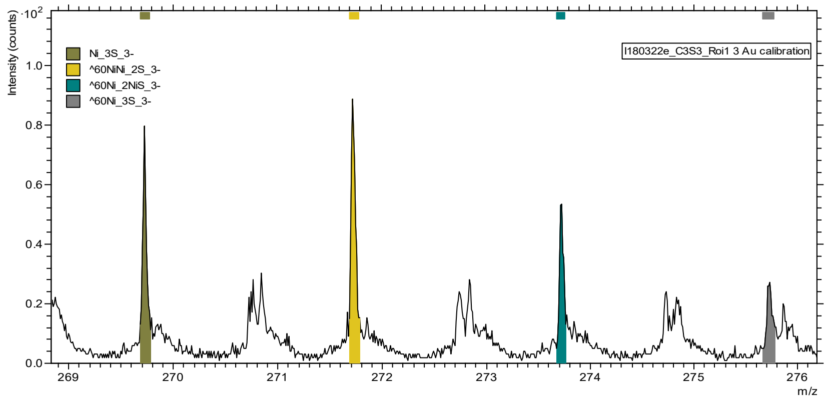
Intensity (counts)
·10^2
Ni_3S_3-
^60NiNi_2S_3-
^60Ni_2NiS_3-
^60Ni_3S_3-
I180322e_C3S3_Roi1 3 Au calibration
m/z

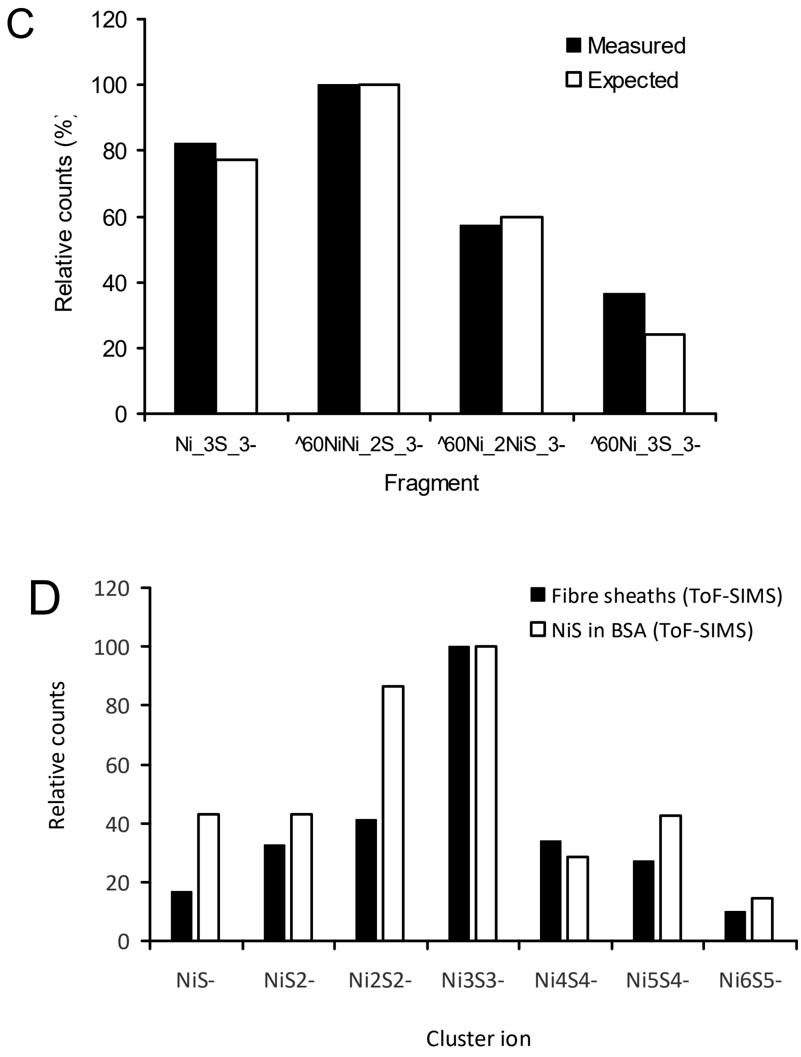


**Supplementary Figure 9.** ToF-SIMS negative mode Ni and S cluster ions from fiber sheaths and a NiS/BSA mixture. A) ToF-SIMS negative mode mass spectrum of a mixture of NiS mineral in BSA. Positions of a selection of important Ni and S cluster ions are indicated. B) Distribution of $Ni_3S_3^-$ isotopomers as a seen in the spectrum of fiber sheaths and C) compared to the expected distribution showing the good fit (similar fits were found for the other $Ni_xS_y$-clusters described, data not shown). D) Distribution of $Ni_xS_y$-cluster ions in fiber sheaths and NiS/BSA mixture (counts were normalized to $Ni_3S_3^-$). Cluster ions with 3 or less Ni atoms were sufficiently mass separated from fragments containing 2 oxygens instead of sulfur such as $Ni_3S_2O_2^-$.

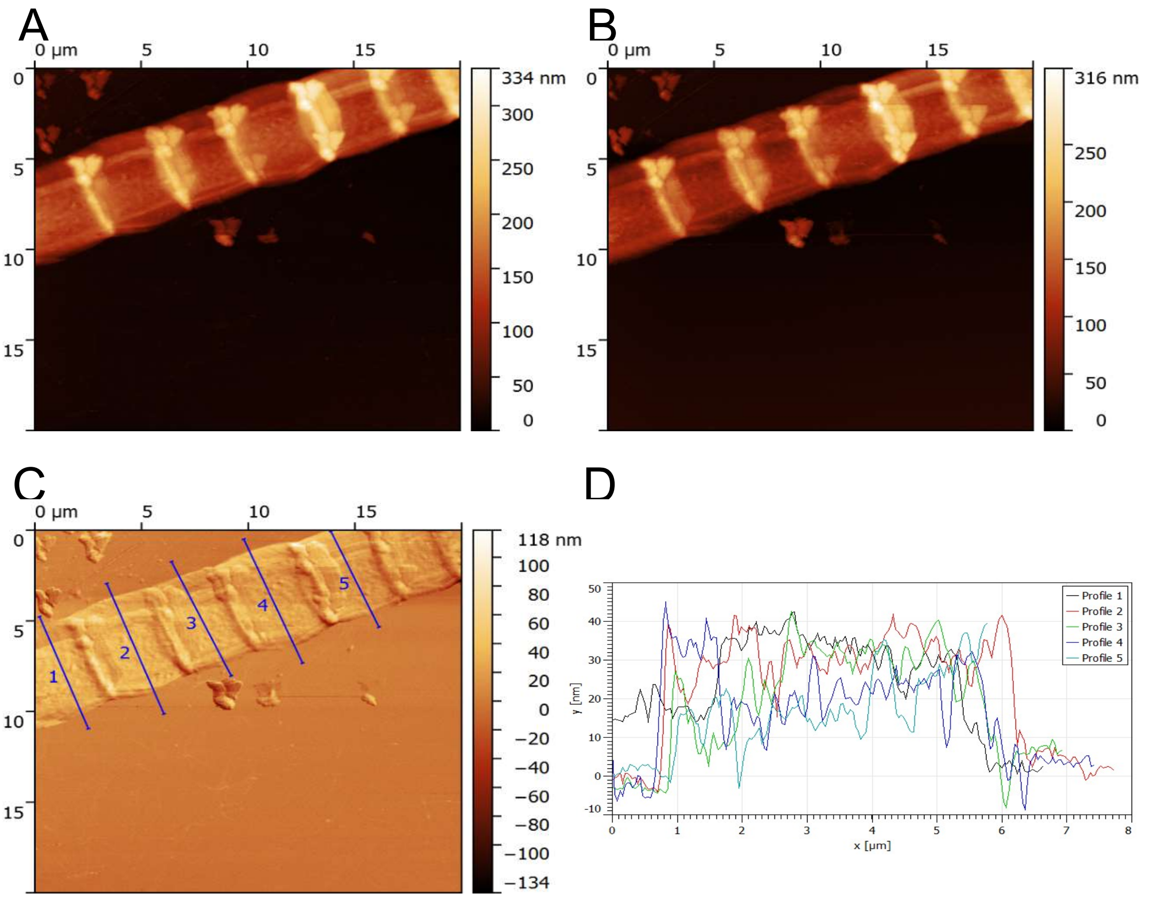


**Supplementary Figure 10.** Example of *in-situ* AFM calibration of ToF-SIMS sputtering depth for fiber sheaths. AFM height map. A) mapping before ToF-SIMS analysis was started. B) mapping after 57 sec of sputtering time (i.e. shortly after the peak in $Ni^{+}$ counts). C) The differential image between (A) and (B) shows the amount of material removed after 57 sec sputtering. Notice shading of the junctions with higher than average sputtering rates on the side and lower than average sputtering rates on the opposite side. D) Height profiles as indicated by the blue lines in panel (C) showing the lateral variation in sputtering depth. Actual sputtering depths were calculated from height profiles as the average difference in height of the fiber sheath (relative to the wafer surface) before and after sputtering (in this example 22.8 ± 3.2 nm).

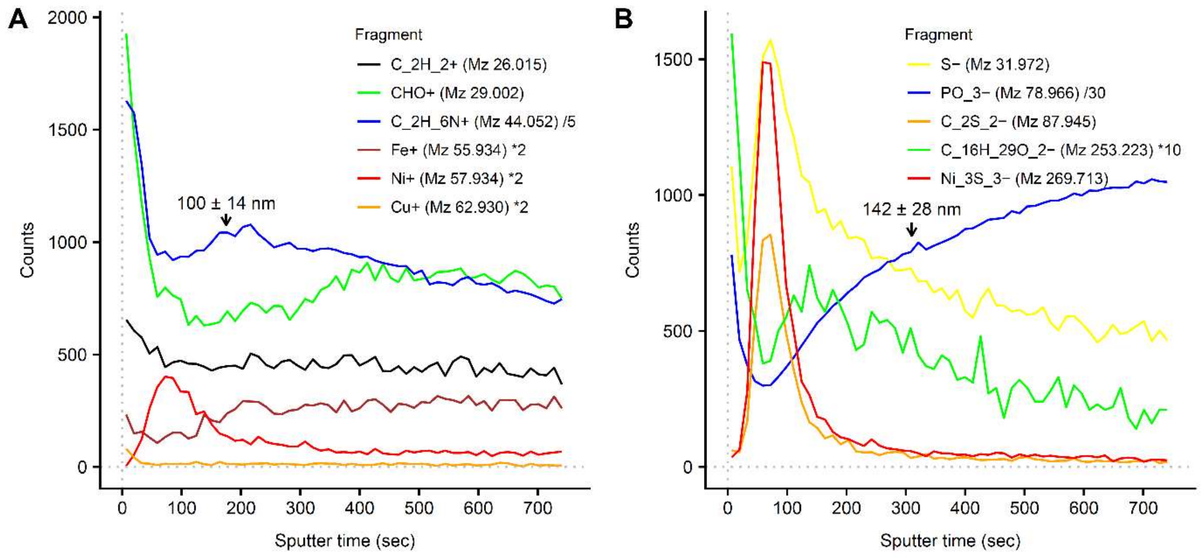


**Supplementary Figure 11.** ToF-SIMS depth profiles of intact cable bacteria demonstrate that the Ni/S group is located in the periplasmic space. Representative ToF-SIMS depth profiles from intact cable bacteria are shown in A) positive mode and B) negative mode. A selection of fragments is shown representing the major biomolecule classes, and counts of individual fragments were scaled to fit all data in a plot. Duplicate runs in both positive and negative modes showed similar profiles. The counts from $Ni_3S_3^-$ are the sum of all $^{58}Ni$ and $^{60}Ni$ isotopologues. The fatty acid fragment ($C_{16}H_{29}O_2^-$) clearly shows two peaks with the first peak at the surface coming most likely from the outer membrane and the broader secondary peak from the cell membrane. Phosphate ($PO_3^-$) also shows a first peak at the surface probably coming from phospholipids and the secondary rise from phospholipids in the cell membrane and poly-phosphate and nucleic acids in the cytoplasm. Arrows indicate sputtering depths as determined with the in-situ AFM in the ToF-SIMS. Average sputtering rate was 0.52 ± 0.05 nm/sec, which places the Ni and S containing peak at approximately 30-40 nm depth in agreement with the expected position of the fibers in intact cable bacteria[2].

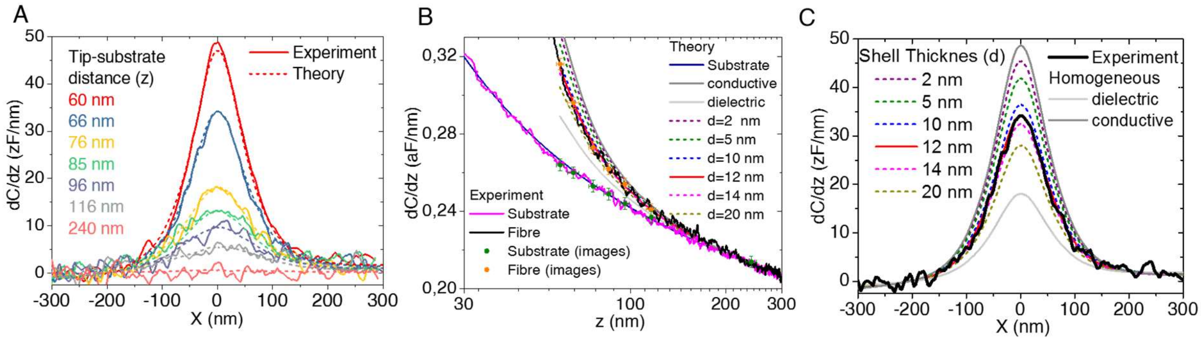


**Supplementary Figure 12.** Additional information on the SDM quantitative analysis shown in Fig. 5 of the main text. A) Capacitance gradient cross-section profiles measured (continuous lines) and calculated (dashed lines) at different tip-substrate distances. The profiles for z=66 nm are those displayed in Fig. 5 of the main text. The parameters used in the calculations are the same as those of Fig. 5 of the main text: H=12.5 μm, W= 3 μm, L=3 μm, l=1 μm, θ=22, R=54 nm, z=60 nm, h=42 nm, w=87 nm, d=12 nm, l=1 μm, $\varepsilon_s = \varepsilon_c = 3$, $\sigma_s = 0$ S/m and $\sigma_c = 2 \cdot 10^3$ S/m. B) Capacitance gradient approach curves measured on a bare part of the substrate (pink continuous line) and on the fiber (black continuous line). The continuous dark blue and red lines correspond to the curves that best fit the experimental results from where the tip geometry (R=54±1 nm, θ=22º±0.5º, $C'_{off}$=112.5±1.5 zF/nm) and shell thickness (d=12±2 nm) have been extracted. The dashed lines represent calculated capacitance gradient approach curves on the fiber for other values of the shell thickness. The continuous dark and grey lines correspond to the homogeneous conductive and dielectric models, respectively. The green and orange symbols correspond to the values measured on the bare substrate and fiber, respectively, on the SDM images at different heights. These values have been used to set the tip-substrate distances of the SDM images. C) Effect of the shell thickness on the calculated capacitance gradient profiles for z=66 nm. The meaning of the lines is the same as in B).

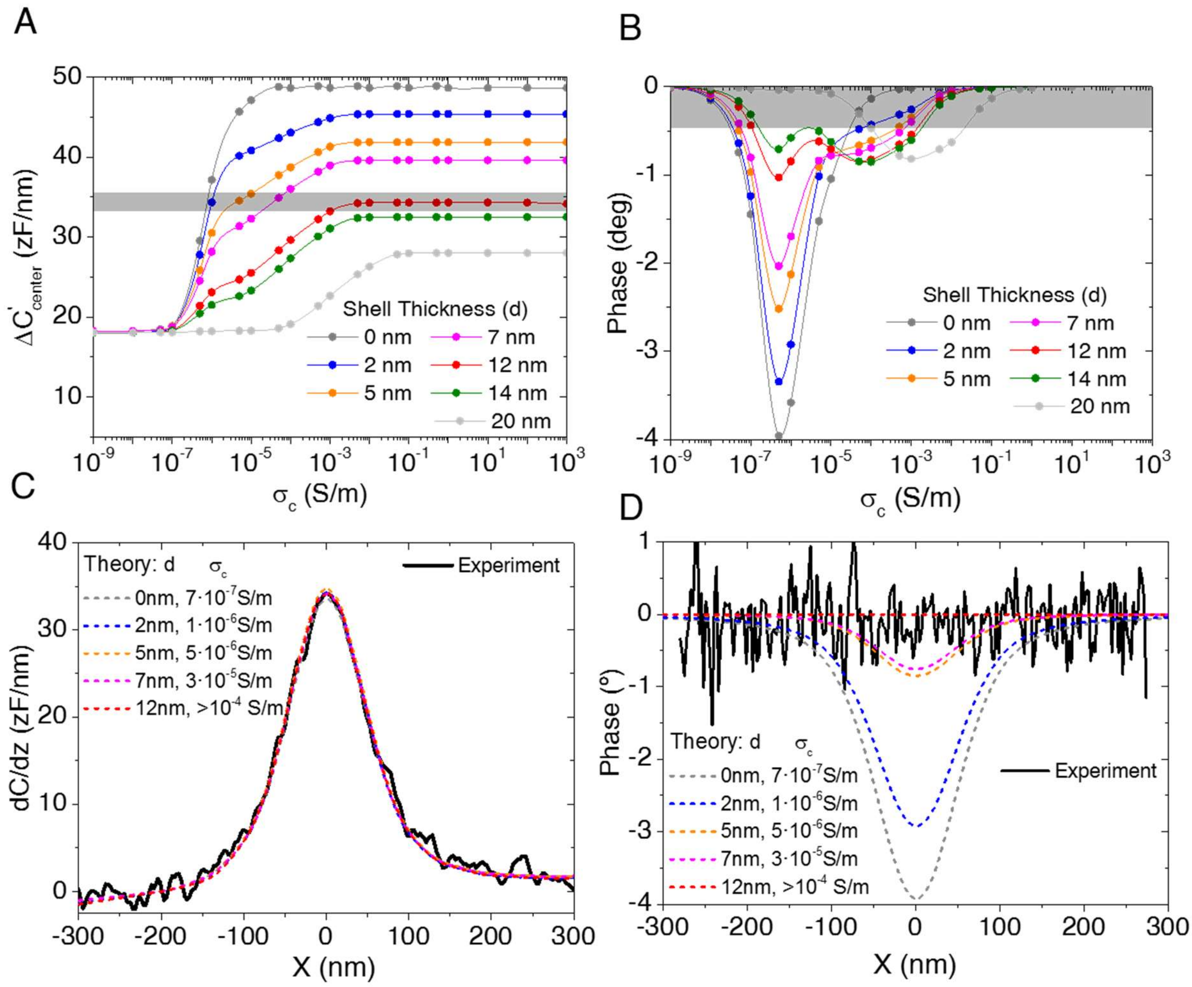


**Supplementary Figure 13.** Additional analysis of the core-shell conductive model used to interpret the SDM measurements shown in Fig. 5 of the main text. A) Amplitude (in capacitance gradient) and B) phase of the electric force contrast calculated for the cylindrical fiber model as a function of the conductivity of the core for different values of the shell thickness. The grey band corresponds to the experimental values extracted from the profiles shown in Fig. 5 of the main text. Calculated capacitance gradient C) and phase D) profiles for the couples of shell thickness-core conductivity that match the experimental measured capacitance gradient contrast in A). The thick lines represent the experimental results (same as in Fig. 5 of the main text). The parameters used in the calculations are the same as those of Fig. 5 of the main text and of Supplementary Figure 12, when not otherwise stated.

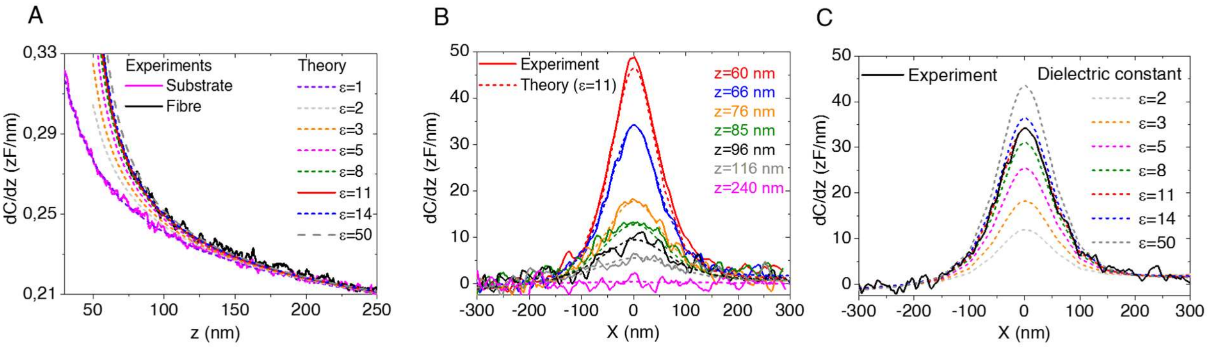


**Supplementary Figure 14.** Analysis of the SDM measurements shown in Fig. 5 of the main text with a homogeneous cylinder dielectric model ($\varepsilon_s = \varepsilon_c = \varepsilon_{eff}$, $\sigma_s = \sigma_c = 0$ S/m). A) Calculated capacitance gradient approach curves on the fiber for different values of the equivalent dielectric constant $\varepsilon_{eff}$ compared to the experimental measured curves on the substrate and fiber (same as in Supplementary Fig 6B, thick lines). The best fit is obtained for $\varepsilon_{eff}$=11±3. B) Calculated capacitance gradient profiles for $\varepsilon_{eff}$=11 for different tip-substrate distances, and comparison with the measured profiles (thick lines, same as in Supplementary Figure 6A). C) Calculated capacitance gradient profiles for z=66 nm and different equivalent dielectric constants $\varepsilon_{eff}$, and comparison with the measured profile (thick line, same as in Fig. 5 of the main text). The parameters used in the calculations are the same as those of Fig. 5 of the main text and of Supplementary Figure 6, when not otherwise stated.

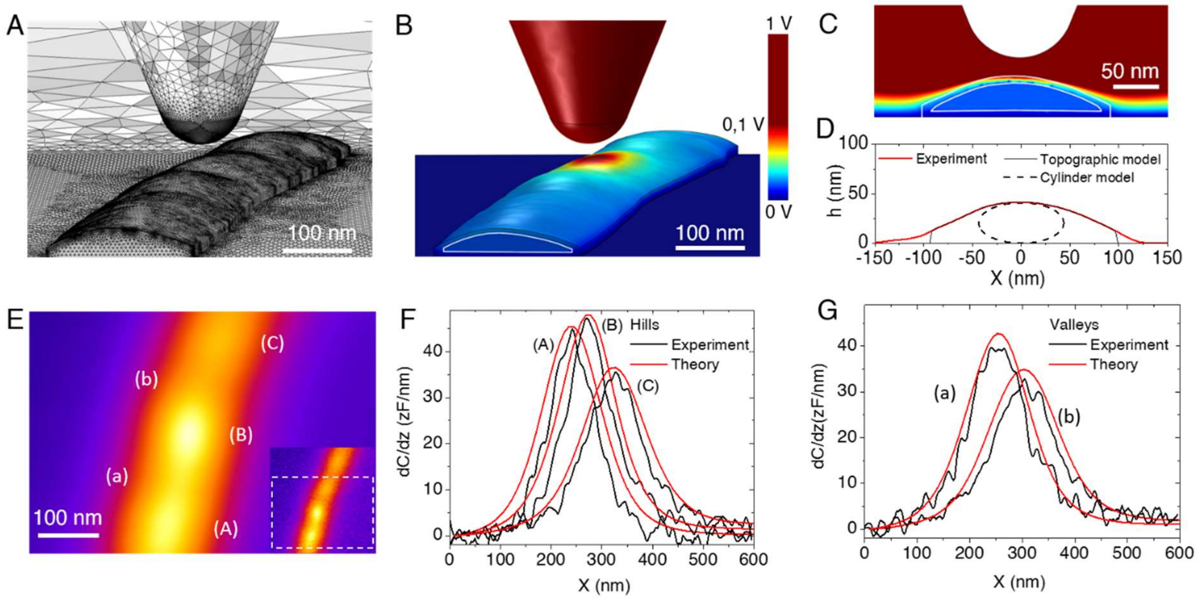


**Supplementary Figure 15.** Analysis of the SDM measurements shown in Fig. 5 of the main text with a geometrical core-shell model reconstructed from the measured topography. A) Detail of the geometry and mesh of the model generated from the measured topography of the fiber (corresponding to the insert in Fig. 5B of the main text). The core-shell structure is defined by assigning a sigmoidal behavior to the dielectric constant and conductivity with plateau representing the shell and core values, respectively. The thickness of the shell corresponds to the center of the sigmoid. B) Example of a calculated electric potential distribution along the fiber for z=66 nm, $\varepsilon_s = \varepsilon_c = 3$, $\sigma_s = 0$ S/m, $\sigma_c = 2 \cdot 10^3$ S/m and d=10 nm. The tip parameters are the same as those in Fig. 5 of the main text. C) Example of a cross-section electric potential distribution corresponding to B). D) Comparison of the cross-section of the measured topography (red line), the topographically reconstructed fiber model (black continuous line) and of the cylinder model (black dashed line). E) Calculated constant height capacitance gradient SDM image by using the model in A) for the parameters in B). The calculated image corresponds to the area enclosed by the dashed line in the insert. F) and G) Comparison of the calculated capacitance gradient profiles (red lines) with the experimental ones (black lines) on the hills (A,B,C) and valleys (a,b) indicated in E), respectively.

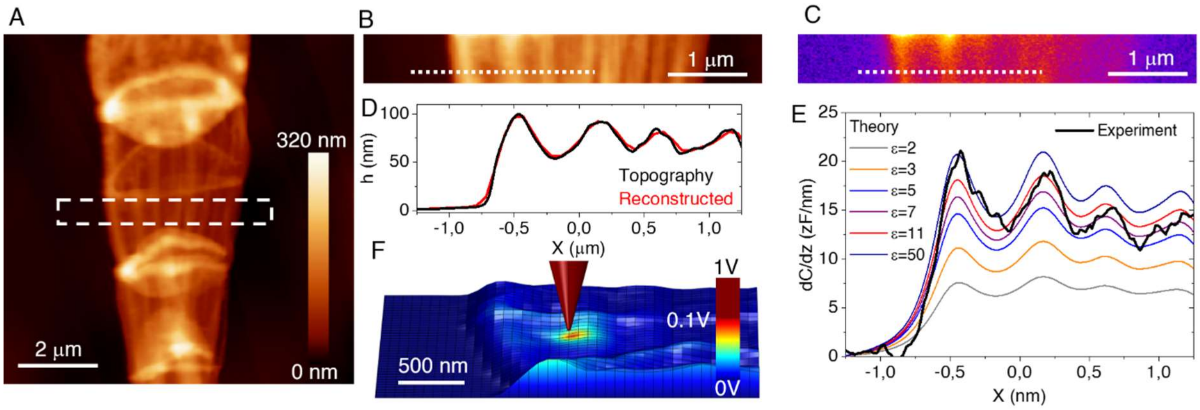


**Supplementary Figure 16.** SDM measurements and analysis on a fiber sheath. A) AFM topographic image of the fiber sheath analyzed. B) and C) AFM topographic and SDM constant height (z=155 nm) images measured on the region enclosed by the dashed rectangle in A). D) and E) Cross-section topographic and capacitance gradient profiles along the dashed lines in B) and C) (thick black lines), respectively. The hills in the image correspond to different fibers. F) Geometrical model reconstructed from the measured topography used in the calculations, with an example of an electric potential distribution overlaid on it. The electrical properties of the fiber sheath have been characterized by an equivalent homogeneous dielectric constant, $\varepsilon_{sheath}$. The tip geometry has been calibrated from a capacitance gradient approach curve on the bare substrate giving R=26±2 nm, θ=22±3º, $C'_{offset}$=109±3 zF/nm. The theoretically predicted capacitance gradient profiles for this model for different values of $\varepsilon_{sheath}$ are shown in E) (thin lines). Most of the hills (fibers) correspond to $\varepsilon_{sheath}$=7−11 in good agreement with the equivalent dielectric constant value found on isolated fibers ($\varepsilon_{eff}$=7−11). For the first hill a higher value is obtained, probably indicating that two fibers are overlaid.

**Supplementary Table 1.** STEM-EDX element compositions for intact cable bacteria and fiber sheaths. Data from two separate imaging sessions are shown (dates in first row).

| | 04-05-17 | | | | 08-08-17 | | | |
|---|---|---|---|---|---|---|---|---|
| | intact cable bacteria N = 3 | | fiber sheath N = 3 | | intact cable bacteria N = 8 | | fiber sheath N = 7 | |
| Elements (atm%) | AVG | SD | AVG | SD | AVG | SD | AVG | SD |
| Carbon | 76.617 | 2.382 | 73.013 | 0.508 | 82.233 | 1.231 | 80.099 | 1.081 |
| Oxygen | 14.370 | 1.185 | 15.880 | 0.322 | 10.854 | 1.394 | 11.319 | 1.205 |
| Nitrogen | 6.133 | 0.250 | 9.283 | 0.224 | 5.411 | 0.547 | 7.333 | 0.477 |
| Phosphorus | 1.120 | 0.607 | 0.663 | 0.100 | 0.833 | 0.167 | 0.736 | 0.150 |
| Sulphur* | 0.510 | 0.035 | 0.350 | 0.225 | 0.483 | 0.078 | 0.329 | 0.023 |
| Zinc | 0.210 | 0.061 | 0.103 | 0.015 | 0.145 | 0.035 | 0.149 | 0.040 |
| Iron | 0.047 | 0.006 | 0.023 | 0.006 | 0.033 | 0.007 | 0.013 | 0.005 |
| Nickel | 0.009 | 0.001 | 0.037 | 0.006 | 0.009 | 0.004 | 0.016 | 0.005 |
| Copper | 0.009 | 0.004 | 0.010 | 0.009 | 0.006 | 0.005 | 0.007 | 0.005 |

*: Sulphur showed interference from a Molybdenum impurity derived most likely from the TEM grid and is therefore uncertain.

## Supplementary references


1. Burdorf, L. D. W. *et al.* Long-distance electron transport occurs globally in marine sediments. *Biogeosciences* **14**, 683–701 (2017).
2. Cornelissen, R. *et al.* The cell envelope structure of cable bacteria. *Front. Microbiol.* **9**, (2018).
3. Bjerg, J. T. *et al.* Long-distance electron transport in individual, living cable bacteria. *Proc. Natl. Acad. Sci.* **115**, 5786–5791 (2018).
4. Eilers, P. H. C. & Boelens, H. F. M. Baseline correction with asymmetric least squares smoothing. *Leiden University Medical Centre Report*. (2005).
5. Fumagalli, L., Esteban-Ferrer, D., Cuervo, A., Carrascosa, J. L. & Gomila, G. Label-free identification of single dielectric nanoparticles and viruses with ultraweak polarization forces. *Nat. Mater.* **11**, 808–816 (2012).
6. Lozano, H. *et al.* Dielectric constant of flagellin proteins measured by scanning dielectric microscopy. *Nanoscale* **10**, 19188–19194 (2018).
7. Lozano, H., Millán-Solsona, R., Fabregas, R. & Gomila, G. Sizing single nanoscale objects from polarization forces. *Sci. Rep.* **9**, 1–12 (2019).
8. Wang, D. *et al.* Interplay between spherical confinement and particle shape on the self-assembly of rounded cubes. *Nat. Commun.* **9**, 2228 (2018).
9. van Aarle, W. *et al.* The ASTRA toolbox: A platform for advanced algorithm development in electron tomography. *Ultramicroscopy* **157**, 35–47 (2015).
10. Gianoncelli, A., Kourousias, G., Merolle, L., Altissimo, M. & Bianco, A. Current status of the TwinMic beamline at Elettra: A soft X-ray transmission and emission microscopy station. *J. Synchrotron Radiat.* **23**, 1526–1537 (2016).

11. Solé, V. A., Papillon, E., Cotte, M., Walter, Ph. & Susini, J. A multiplatform code for the analysis of energy-dispersive X-ray fluorescence spectra. *Spectrochim. Acta Part B At. Spectrosc.* **62**, 63–68 (2007).
12. Polerecky, L. *et al.* Look@NanoSIMS - a tool for the analysis of nanoSIMS data in environmental microbiology. *Environ. Microbiol.* **14**, 1009–1023 (2012).
13. Meysman, F. J. R. *et al.* A highly conductive fibre network enables centimetre-scale electron transport in multicellular cable bacteria. *Nat. Commun.* **10**, 1–8 (2019).
14. Baugh, L. *et al.* Probing the orientation of surface-immobilized protein G B1 using ToF-SIMS, sum frequency generation, and NEXAFS spectroscopy. *Langmuir* **26**, 16434–16441 (2010).
15. Goacher, R. E., Jeremic, D. & Master, E. R. Expanding the library of secondary ions that distinguish lignin and polysaccharides in time-of-flight secondary ion mass spectrometry analysis of wood. *Anal. Chem.* **83**, 804–812 (2011).
16. Wei, W. *et al.* Characterization of syntrophic Geobacter communities using ToF-SIMS. *Biointerphases* **12**, 05G601 (2017).
17. Lebec, V., Boujday, S., Poleunis, C., Pradier, C.-M. & Delcorte, A. Time-of-flight secondary ion mass spectrometry investigation of the orientation of adsorbed antibodies on SAMs correlated to biorecognition tests. *J. Phys. Chem. C* **118**, 2085–2092 (2014).
18. Ding, Y. *et al.* In Situ Molecular Imaging of the Biofilm and Its Matrix. *Anal. Chem.* **88**, 11244–11252 (2016).
19. Lee, C.-Y., Harbers, G. M., Grainger, D. W., Gamble, L. J. & Castner, D. G. Fluorescence, XPS, and TOF-SIMS Surface Chemical State Image Analysis of DNA Microarrays. *J. Am. Chem. Soc.* **129**, 9429–9438 (2007).

20. May, C. J., Canavan, H. E. & Castner, D. G. Quantitative X-ray Photoelectron Spectroscopy and Time-of-Flight Secondary Ion Mass Spectrometry Characterization of the Components in DNA. *Anal. Chem.* **76**, 1114–1122 (2004).
21. Lovering, A. L., Safadi, S. S. & Strynadka, N. C. J. Structural perspective of peptidoglycan biosynthesis and assembly. *Annu. Rev. Biochem.* **81**, 451–478 (2012).
22. Geerlings, N. M. J., Zetsche, E.-M., Hidalgo Martinez, S., Middelburg, J. J. & Meysman, F. J. R. Mineral formation induced by cable bacteria performing long-distance electron transport in marine sediments. *Biogeosciences Discuss.* 1–35 (2018) doi:10.5194/bg-2018-444.
23. Franquet, A. *et al.* Self focusing SIMS: Probing thin film composition in very confined volumes. *Appl. Surf. Sci.* **365**, 143–152 (2016).
24. Feld, H., Rading, D., Leute, A. & Benninghoven, A. Comparative investigations of the secondary ion emission of metal complexes under MeV and keV ion bombardment. *Org. Mass Spectrom.* **28**, 841–852 (1993).
25. El Nakat, J. H., Dance, I. G., Fisher, K. J., Rice, D. & Willett, G. D. Laser-ablation FTICR (Fourier-transform ICR) mass spectrometry of metal sulfides: gaseous anionic nickel-sulfur [NixSy] clusters. *J. Am. Chem. Soc.* **113**, 5141–5148 (1991).
26. Jones, T. B. *Electromechanics of particles*. (Cambridge University Press, 1995).
27. Cuervo, A. *et al.* Direct measurement of the dielectric polarization properties of DNA. *Proc. Natl. Acad. Sci.* **111**, E3624–E3630 (2014).
28. Dols-Perez, A., Gramse, G., Calò, A., Gomila, G. & Fumagalli, L. Nanoscale electric polarizability of ultrathin biolayers on insulating substrates by electrostatic force microscopy. *Nanoscale* **7**, 18327–18336 (2015).
29. Checa, M. *et al.* Mapping the dielectric constant of a single bacterial cell at the nanoscale with scanning dielectric force volume microscopy. *Nanoscale* **11**, 20809–20819 (2019).

30. Fabregas, R. & Gomila, G. Dielectric nanotomography based on electrostatic force microscopy: A numerical analysis. *J. Appl. Phys.* **127**, 024301 (2020).

31. Gray, H. B. & Winkler, J. R. Electron flow through metalloproteins. *Biochim. Biophys. Acta BBA - Bioenerg.* **1797**, 1563–1572 (2010).

32. Fischer, H., Polikarpov, I. & Craievich, A. F. Average protein density is a molecular-weight-dependent function. *Protein Sci.* **13**, 2825–2828 (2004).
