# Supplementary material for: Efficient long-range conduction in cable bacteria through nickel protein wires
Source: Nat Commun. 2021 Jun 28;12:3996. doi: 10.1038/s41467-021-24312-4 (PMC8238962; doi:10.1038/s41467-021-24312-4)
Supplement: Supplementary file 3 — Description of Additional Supplementary Files [file 41467_2021_24312_MOESM3_ESM.pdf]

Description of Additional Supplementary Files

File Name: Supplementary Data 1
Description: Identified fragment ions in positive mode ToF-SIMS of fiber sheaths extracted from cable bacteria.

File Name: Supplementary Data 2
Description: Identified fragment ions in negative mode ToF-SIMS of fiber sheaths extracted from cable bacteria.

File Name: Supplementary Movie 1
Description: 3D tomographic reconstruction of the freeze dried fiber sheath in Fig. 1A
