# Supplementary material for: Efficient long-range conduction in cable bacteria through nickel protein wires
Source: Nat Commun. 2021 Jun 28;12:3996. doi: 10.1038/s41467-021-24312-4 (PMC8238962; doi:10.1038/s41467-021-24312-4)
Supplement: Supplementary file 7 — Reporting Summary [file 41467_2021_24312_MOESM7_ESM.pdf]

nature research

Corresponding author(s): H.T.S. Boschker and Filip J.R. Meysman

Last updated by author(s): May 31, 2021

# Reporting Summary

Nature Research wishes to improve the reproducibility of the work that we publish. This form provides structure for consistency and transparency in reporting. For further information on Nature Research policies, see our Editorial Policies and the Editorial Policy Checklist.

## Statistics

For all statistical analyses, confirm that the following items are present in the figure legend, table legend, main text, or Methods section.

| n/a | Confirmed | |
|---|---|---|
| ☐ | ☒ | The exact sample size ($n$) for each experimental group/condition, given as a discrete number and unit of measurement |
| ☐ | ☒ | A statement on whether measurements were taken from distinct samples or whether the same sample was measured repeatedly |
| ☐ | ☒ | The statistical test(s) used AND whether they are one- or two-sided<br>*Only common tests should be described solely by name; describe more complex techniques in the Methods section.* |
| ☒ | ☐ | A description of all covariates tested |
| ☒ | ☐ | A description of any assumptions or corrections, such as tests of normality and adjustment for multiple comparisons |
| ☐ | ☒ | A full description of the statistical parameters including central tendency (e.g. means) or other basic estimates (e.g. regression coefficient) AND variation (e.g. standard deviation) or associated estimates of uncertainty (e.g. confidence intervals) |
| ☐ | ☒ | For null hypothesis testing, the test statistic (e.g. $F$, $t$, $r$) with confidence intervals, effect sizes, degrees of freedom and $P$ value noted<br>*Give P values as exact values whenever suitable.* |
| ☒ | ☐ | For Bayesian analysis, information on the choice of priors and Markov chain Monte Carlo settings |
| ☒ | ☐ | For hierarchical and complex designs, identification of the appropriate level for tests and full reporting of outcomes |
| ☒ | ☐ | Estimates of effect sizes (e.g. Cohen's $d$, Pearson's $r$), indicating how they were calculated |

*Our web collection on statistics for biologists contains articles on many of the points above.*

## Software and code

Policy information about availability of computer code

| | |
|---|---|
| Data collection | SDM: COMSOL Multiphysics 5.3, Matlab 2019 |
| Data analysis | STEM tomography: Astra toolbox v1.8<br>ToF-SIMS: SurfaceLab 5, PCA R package FactoMineR v2.4<br>Raman: R v3.5.1, RStudio v1.2.1335, packages Baseline v1.3-1, hyperSpec v0.990<br>STEM-EDX: Bruker ESPRIT v2<br>LEXRF: PyMCA<br>Nano-SIMS: Look@NanoSIMS 2018-05-20<br>SDM: Matlab 2019<br>AMF: Gwyddion 2.50<br>Statistics: R function wilcox.test<br>Figures: R packages ggplot2 v3.3.3, ggrepel v0.9.1, cowplot v1.1.1, |

For manuscripts utilizing custom algorithms or software that are central to the research but not yet described in published literature, software must be made available to editors and reviewers. We strongly encourage code deposition in a community repository (e.g. GitHub). See the Nature Research guidelines for submitting code & software for further information.

## Data

Policy information about availability of data

All manuscripts must include a data availability statement. This statement should provide the following information, where applicable:

- Accession codes, unique identifiers, or web links for publicly available datasets
- A list of figures that have associated raw data
- A description of any restrictions on data availability

Detailed ToF-SIMS and SDM results and discussion are available in Supplementary Notes 1-2. Source data are provided for Figures 1-5, Supplementary Figures 1-4 and 11 and Supplementary Table 1. Other relevant data will be made available by the authors upon request.

# Field-specific reporting

Please select the one below that is the best fit for your research. If you are not sure, read the appropriate sections before making your selection.

- [x] Life sciences
- [ ] Behavioural & social sciences
- [ ] Ecological, evolutionary & environmental sciences

For a reference copy of the document with all sections, see nature.com/documents/nr-reporting-summary-flat.pdf

# Life sciences study design

All studies must disclose on these points even when the disclosure is negative.

| | |
|---|---|
| Sample size | A priori sample size determination was not done. Experiments and chemical imaging on bacterial filaments were replicated until statistical parameters (e.g. standard error of the mean) were stable. |
| Data exclusions | No data were excluded from the analysis |
| Replication | All laboratory experiments and chemical imaging reported were replicated at least twice to test reproducibility. Replication was successful in each case as reported in the paper. The only experiment that could not be replicated was the SDM imaging of a single fiber, as the detachment of an intact single fiber from a cable bacterium fiber sheath is a rare event (and only one such sample was available). However, the SDM imaging served to confirm the existing core/shell model hypothesis that was already put forward by the Tof-SIMS and other chemical imaging techniques. The the SDM imaging was not used to bring forward a new hypothesis. Furthermore, SDM was also applied to a double fiber detached from a fiber sheath and an intact fiber sheath as described on Supplementary Note 2, which are both in agreement with the single fiber SDM. |
| Randomization | Cable bacteria filaments were randomly picked from sediments and assigned to treatment groups. |
| Blinding | Blinding was not used. All treatment groups were analyzed using standardized protocols. |

# Reporting for specific materials, systems and methods

We require information from authors about some types of materials, experimental systems and methods used in many studies. Here, indicate whether each material, system or method listed is relevant to your study. If you are not sure if a list item applies to your research, read the appropriate section before selecting a response.

### Materials & experimental systems

| n/a | Involved in the study | |
|---|---|---|
| x | | Antibodies |
| x | | Eukaryotic cell lines |
| x | | Palaeontology and archaeology |
| x | | Animals and other organisms |
| x | | Human research participants |
| x | | Clinical data |
| x | | Dual use research of concern |

### Methods

| n/a | Involved in the study | |
|---|---|---|
| x | | ChIP-seq |
| x | | Flow cytometry |
| x | | MRI-based neuroimaging |
